# Technical Note: 4D cone‐beam CT reconstruction from sparse‐view CBCT data for daily motion assessment in pencil beam scanned proton therapy (PBS‐PT)

**DOI:** 10.1002/mp.14521

**Published:** 2020-10-24

**Authors:** Lydia A. den Otter, Kuanling Chen, Guillaume Janssens, Arturs Meijers, Stefan Both, Johannes A. Langendijk, Lane R. Rosen, Hsinshun T. Wu, Antje‐Christin Knopf

**Affiliations:** ^1^ Department of Radiation Oncology University Medical Center Groningen University of Groningen Groningen 9713 GZ The Netherlands; ^2^ Department of Radiation Oncology Willis‐Knighton Cancer Center Shreveport LA USA; ^3^ Ion Beam Applications, Research and Development Louvain‐la‐Neuve Belgium

**Keywords:** 4D‐CBCT, image reconstruction, inter‐fractional motion, Lung cancer, pencil beam scanning, proton therapy

## Abstract

**Purpose:**

The number of pencil beam scanned proton therapy (PBS‐PT) facilities equipped with cone‐beam computed tomography (CBCT) imaging treating thoracic indications is constantly rising. To enable daily internal motion monitoring during PBS‐PT treatments of thoracic tumors, we assess the performance of **M**otion‐**A**ware **R**ec**O**nstructi**O**n method using **S**patial and **Te**mporal **R**egularization (MA‐ROOSTER) four‐dimensional CBCT (4DCBCT) reconstruction for sparse‐view CBCT data and a realistic data set of patients treated with proton therapy.

**Methods:**

Daily CBCT projection data for nine non‐small cell lung cancer (NSCLC) patients and one SCLC patient were acquired at a proton gantry system (IBA Proteus® One). Four‐dimensional CBCT images were reconstructed applying the MA‐ROOSTER and the conventional phase‐correlated Feldkamp‐Davis‐Kress (PC‐FDK) method. Image quality was assessed by visual inspection, contrast‐to‐noise ratio (CNR), signal‐to‐noise ratio (SNR), and the structural similarity index measure (SSIM). Furthermore, gross tumor volume (GTV) centroid motion amplitudes were evaluated.

**Results:**

Image quality for the 4DCBCT reconstructions using MA‐ROOSTER was superior to the PC‐FDK reconstructions and close to FDK images (median CNR: 1.23 [PC‐FDK], 1.98 [MA‐ROOSTER], and 1.98 [FDK]; median SNR: 2.56 [PC‐FDK], 4.76 [MA‐ROOSTER], and 5.02 [FDK]; median SSIM: 0.18 [PC‐FDK vs FDK], 0.31 [MA‐ROOSTER vs FDK]). The improved image quality of MA‐ROOSTER facilitated GTV contour warping and realistic motion monitoring for most of the reconstructions.

**Conclusion:**

MA‐ROOSTER based 4DCBCTs performed well in terms of image quality and appear to be promising for daily internal motion monitoring in PBS‐PT treatments of (N)SCLC patients.

## 1 INTRODUCTION

In recent years, cone‐beam computed tomography (CBCT) acquisition has become available at pencil beam scanned proton therapy (PBS‐PT) facilities with the main purpose of daily positioning and anatomy verification.^1^ As PBS‐PT has also been extended for an increasing number of PT facilities totreatment of moving targets, daily motion monitoring in treatment position would be of added value. This would require a four‐dimensional (4D) reconstruction method where currently only three‐dimensional cone‐beam computed tomography (3DCBCT) reconstruction software is commercially available for proton CBCT installations. In addition, to keep the imaging dose and the imaging time at the gantry as low as possible, 4DCBCT reconstruction methods are preferred that only require projection data acquired during 3DCBCT acquisition. The conventionally used phase‐correlated algorithm of Feldkamp‐Davis‐Kress (PC‐FDK; Ref. [2]) suffers from undersampling artifacts when using a limited amount of projections. One alternative is the McKinnon‐Bates (MKB) or the recently published modified MKB method, which starts with a motion‐blurred FDK prior image and PC‐FDK data. It creates motion‐encoded difference projections that are reconstructed and added to the well‐sampled FDK prior image to create the higher quality 4DCBCTs.^3^, ^4^ Other advanced 4D reconstruction methods have been reported that work well for few projection data by applying for example regularization between neighboring reconstructed phases or using prior image constrained compressed sensing (PICCS) reconstruction.^5^, ^6^, ^7^ Furthermore, motion‐compensated methods have proven to successfully improve the image quality.^8^, ^9^, ^10^, ^11^ Combinations of the different techniques have shown great potential in the last years.^12^, ^13^, ^14^ Recently, the SPARE challenge was performed where several reconstruction methods for 1‐min acquisitions were compared for image quality and a common framework for evaluation of reconstruction methods was built.^15^ The method best performing in this challenge was the so‐called **M**otion‐**A**ware **R**ec**O**nstructi**O**n method using **S**patial and **Te**mporal **R**egularization (MA‐ROOSTER), introduced by Mory et al.^16^ For phantom data with known motion amplitudes, this method has shown to be promising to implement for scanned proton gantry systems.^16^

Only one published study investigated the potential of using (sparse‐view) 4DCBCTs for proton therapy treatments specifically.^17^ This was done using phantom CBCT data acquired at a medical linear accelerator that was reconstructed using an iterative reconstruction method based on total variation regularization.^17^

This technical note reports on the potential of using the advanced reconstruction method MA‐ROOSTER for creating 4DCBCTs from sparse‐view clinical CBCT data. This was done for a representative proton therapy data set of (non‐)small cell lung cancer ([N]SCLC) patients. 4DCBCT image quality and the internal tumor motion for its suitability for daily internal motion monitoring were assessed.

## 2 MATERIALS AND METHODS

### 2.A Patient data

The data set consisted of nine patients with NSCLC and one patient with SCLC who were treated with PBS‐PT with curative intent. For each patient, daily CBCT projection data were acquired at an IBA Proteus® One proton gantry system. The first ten fractions of CBCT projections were used in this study to show the feasibility of 4DCBCT reconstruction including daily variations in image quality. The integrated CBCT system has a source‐to‐axis distance of 100 cm and source‐to‐imager distance of 155 cm. Image acquisition settings were a field‐of‐view of 25 cm, 110 kVp, 25 mA, and 6.4 CTDI_weighted_ (mGy). Each projection had a resolution of 1441 by 1440, with image plane pixel spacing of 0.3 mm. Acquisition was performed during a ≈220‐degree arc rotation, average gantry speed of 3 degrees per second, and a total scan time of ≈1.2 min. Per set a total of 515 projections were acquired. The ten patients were divided in two groups for image quality assessment, based on the presence of metallic artifacts in the images. Patients #1 and #2 had a Port‐a‐Cath, patient #3 had a trachea stoma, and patient #4 had a cervical fusion plate. Patient #5 had surgical clips in the breast and axillary region as this patient was treated for breast cancer. Patient #1 suffered also from high‐density metastatic spinal lesions. Additionally, the simulation 4D‐ and 3DCT scans with clinical structure sets were available. Five out of ten patients received one repeated 4DCT scan after 1 or 2 weeks of treatment, because of tumor volume shrinkage (pts. #1, #4, #9, and #10) and pleural effusion (pt. #7).

### 2.B 4DCBCT reconstruction

The reconstruction method of MA‐ROOSTER is implemented in the Reconstruction ToolKit (RTK) library, an open‐source C++ software based on the Insight Toolkit (ITK).^18^ Preprocessing was performed in the MATLAB (MathWorks, Natick, MA) based open‐source collaboration software REGGUI (https://openreggui.org/). In REGGUI, deformable image registration (DIR) was performed (diffeomorphic morphons algorithm, developed by Janssens et al.^19^) between the simulation 4DCT phases to compute the 4D deformable vector fields (DVF). The 4D DVFs are used during temporal regularization of the MA‐ROOSTER method. The complete workflow was executed on a Linux machine with Intel Xeon CPU E5‐2650 processor v4 (2.4 GHz), 24 cores, and 48 hyper‐threads. It has 64 GB DDR4 memory and two GPUs (GTX 1080 Ti) installed in parallel. An NVIDIA tesla driver (vs 418.87.1) and CUDA (version 10.1) were utilized for fast GPU computations. The same parameter settings were applied as described by Mory et al.^16^ for spatial and temporal regularization. These settings were found to be the most optimal for our patients for removing streak artifacts and at the same time prevent image blurring by over‐regularization. To compare the image quality and motion monitoring performance of MA‐ROOSTER reconstructions, all data were additionally reconstructed using the conventionally used PC‐FDK method. PC‐FDK is not considered to be equally comparable in performance; however, it shows the baseline image quality when using a phase‐correlated method. Also, FDK 3DCBCT images were created to show the image quality of FDK for ample projections. Both FDK and PC‐FDK are available in the RTK library. Table I shows the other parameters and settings for MA‐ROOSTER and PC‐FDK. All 4DCBCTs were reconstructed in ten single respiratory phases and binned following the internal respiratory signal of the diaphragm region that was semi‐automatically extracted using the Amsterdam Shroud method.^20^ The size of the reconstructed images depended on the patient’s size and varied between 28–30 cm × 22–28 cm × 20–28 cm.

**Table I:** Parameters and settings of four‐dimensional cone‐beam computed tomography (4DCBCT) reconstruction.

Parameters	MA‐ROOSTER	PC‐FDK
Nr. of phases	10	10
Voxel spacing (mm)	2 × 2 × 2	2 × 2 × 2
Hann windowing		0.5
Projection padding		0.1
Number of iterations	10	
Nr. of nested CG iterations	4	
Nr. of nested TV iterations	10	
Temporal regularization (γ_time_)	0.0002	
Spatial regularization (γ_space_)	0.00005	

CG = conjugate gradient; TV = total variation.

### 2.C Deformable image registration for contour warping

After reconstruction, the 4DCBCT images were imported in RayStation (RaySearch Laboratories, Stockholm, Sweden), together with the 3DCBCT images, the simulation and repeat (4D)CT scans and the clinical structure sets. The simulation CTs were rigidly registered to all the 4DCBCT and 4DCT phases. Afterward, the gross tumor volume (GTV) contours of the simulation CTs were deformably warped to all the 4DCBCT and 4DCT phases using the ANACONDA algorithm available in RayStation.^21^ A different DIR algorithm was chosen for contour warping compared to the DIR (diffeomorphic morphons) used during the preprocessing phase of the MA‐ROOSTER reconstruction to avoid a bias toward the MA‐ROOSTER reconstructions in motion estimation.

### 2.D Image quality metrics

Image quality was assessed for all phases of the 4DCBCTs and the FDK images using three measures. The first two are the contrast‐to‐noise ratio (CNR) and signal‐to‐noise ratio (SNR), calculated as follows:

(1) $$CNR=\frac{\left|\mu_{\mathrm{GTV}}‐\mu_{\mathrm{Lung}}\right|}{\left(\sigma_{\mathrm{Lung}}\right)}$$

(2) $$SNR=\left[\frac{\mu_{\mathrm{Lung}}}{\sigma_{\mathrm{Lung}}}\right]$$

where $\mu_{\mathrm{GTV}}$ represents the average attenuation value in Hounsfield units (HU) measured in the GTV. $\mu_{\mathrm{Lung}}$ was determined as average HU in a 2–10 mm margin surrounding the GTV. The margin was adapted to create a region of comparable volume size as the GTV. $\sigma_{\mathrm{Lung}}$ represents the standard deviations of the HU measured in the lung margin surrounding the GTV. The contoured GTV often includes lung tissue in some extent, and therefore differs in tissue heterogeneity between patients. To determine the signal‐to‐noise ratio, we chose to use the surrounding and less heterogeneous lung tissue region for calculation. The third is the Structural Similarity Index Measure (SSIM), which evaluates a combination of image luminance, contrast, and structure. The SSIM is available in the MATLAB environment.^22^ The comparison with SSIM was performed between each phase of the PC‐FDK or MA‐ROOSTER full images and the FDK full images.

### 2.E Motion analysis

**Fig. 1:**
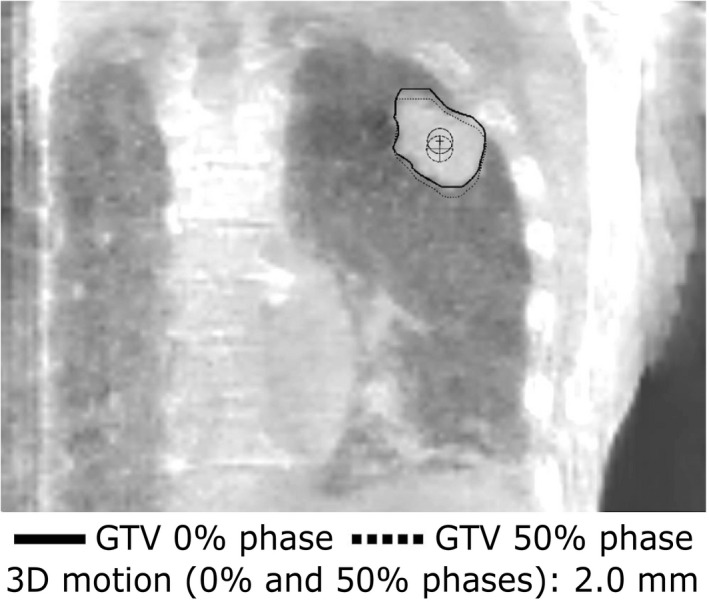
Coronal view of a contoured gross tumor volume and both centroid positions for the 0% (solid line) and 50% (dotted line) phase. The corresponding three‐dimensional vector motion is noted additionally.

The GTV centroid positions were calculated for all phases in order to assess the motion amplitudes (Fig. 1). For each combination of two phases, the distance was calculated in three directions (superior–inferior [SI], anterior–posterior [AP], right–left [RL]). For each combination, the total 3D displacement vector length was calculated using the three calculated distances: $\surd \left(SI^{2}+AP^{2}+RL^{2}\right)$. Finally, the largest calculated displacement was selected to be the full 3D tumor motion amplitude.

## 3 RESULTS

### 3.A 4D image quality assessment

**Fig. 2:**
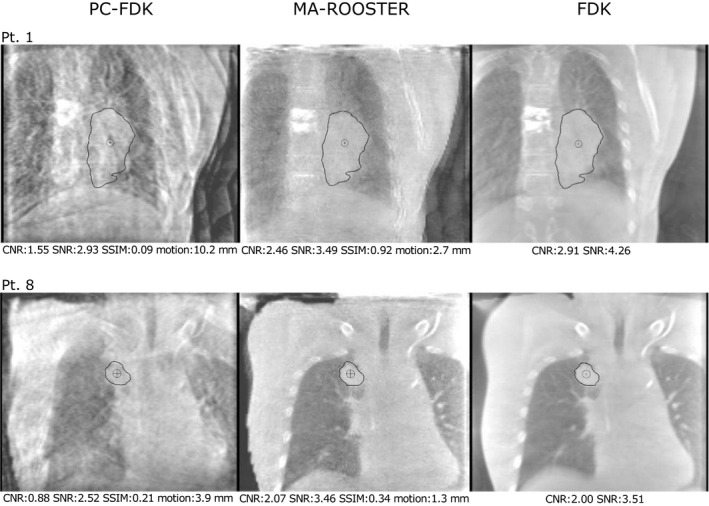
Visual comparison (coronal views) of one of the reconstructed phases of the MA‐ROOSTER method compared to the same phase of the PC‐FDK and FDK reconstructed images for two patients with differing image quality (pt. #1 suffering from additional image distortion). Quality measures for these reconstructions are shown below the figures. These include the contrast‐to‐noise ratio (CNR), signal‐to‐noise ratio (SNR), and structural similarity index measures (SSIM). The centroid motion calculated between all phases for the shown treatment fractions of the two patients are noted additionally.

**Fig. 3:**
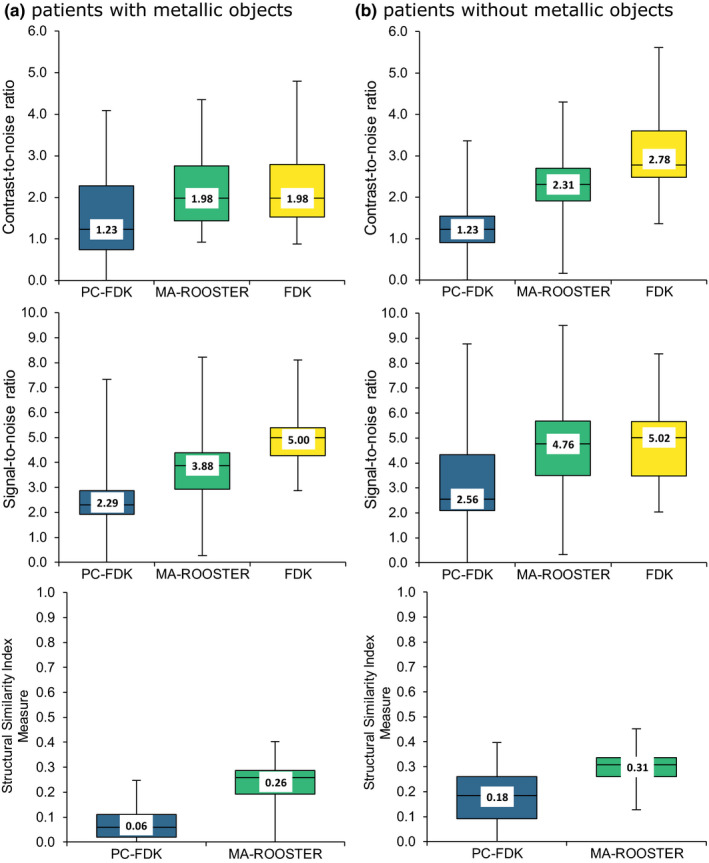
Image quality measures compared for the PC‐FDK, MA‐ROOSTER, and FDK image reconstructions, assessed for two groups of five patients with (a) and without (b) metallic objects that caused image distortions. The box plots are shown together with the median values. The lower box ranges from the first quartile to median, the upper box ranges from median to the third quartile. The lower and upper whiskers range from the first quartile and third quartile to the minimum and maximum values, respectively. [Color figure can be viewed at wileyonlinelibrary.com]

Visual inspection of the images as well as the image quality measures revealed that the MA‐ROOSTER reconstructed images were close in image quality to the FDK images (Figs. 2 and 3). This was more apparent for the patient group without metallic objects, as the metal hampered the image quality of both MA‐ROOSTER and PC‐FDK reconstructions (pt. #1, Figs. 2 and 3).

### 3.B Motion evaluation

**Fig. 4:**
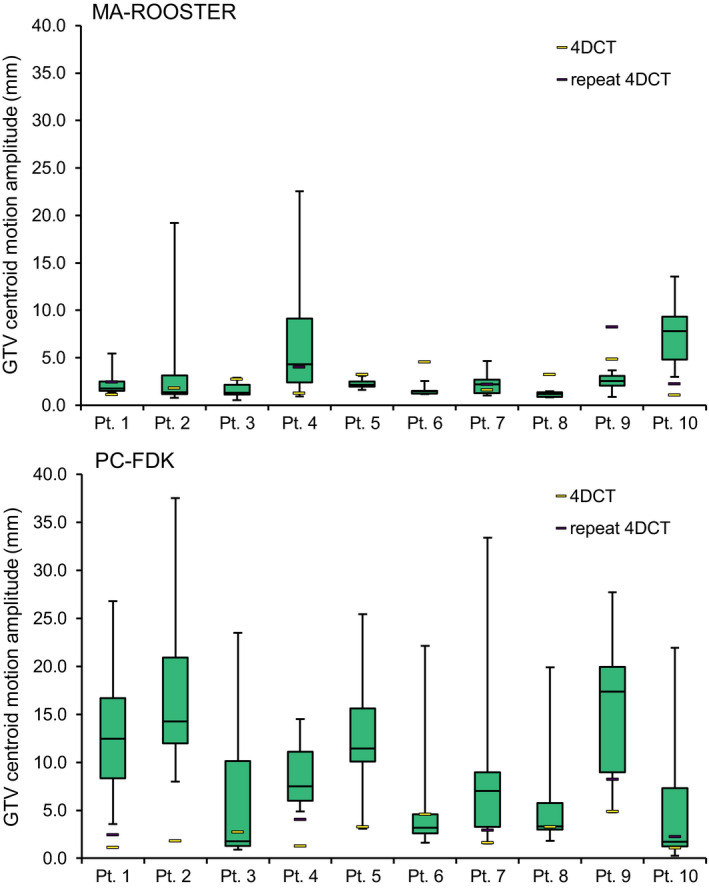
Range of gross tumor volume centroid motion amplitudes for each patient, measured on the daily four‐dimensional cone‐beam computed tomography (4DCBCT) scans (box plots), simulation 4DCTs, and repeat 4DCTs (5/10 patients). The box plots depict the first quartile to median (lower box) and median to third quartile (upper box). The lower and upper whiskers range from the first quartile and third quartile to the minimum and maximum values, respectively. [Color figure can be viewed at wileyonlinelibrary.com]

GTV centroid motion amplitudes of the 4DCBCTs after deformable contour warping are shown in Fig. 4. For the ten patients, median centroid motion amplitudes varied between 1.2 and 7.8 mm following the MA‐ROOSTER method and varied between 1.7 and 17.3 mm for the PC‐FDK reconstructions. The found motion amplitudes extended up till 37.5 mm for the PC‐FDK method, as a result from the often poor image quality. Large motion amplitudes were also found for the MA‐ROOSTER reconstructed images of patients #2 and #4, due to metallic implants hampering image quality. Pt. #10 showed a large decrease in tumor volume, which was also challenging for MA‐ROOSTER. GTV centroid motion amplitudes of the simulation and repeat 4DCTs (pts. #1, #4, #7, #9, and #10) are additionally shown in Fig. 4. The MA‐ROOSTER reconstructions were closer to the found motion of the 4DCTs compared to the PC‐FDK reconstructions.

## 4 DISCUSSION

This technical note showed that MA‐ROOSTER is a feasible method to reconstruct 4DCBCT images in a clinical setting with sparse‐view CBCT projection data acquired at a scanned proton gantry. MA‐ROOSTER resulted in improved image quality compared to the conventionally used PC‐FDK reconstruction approach, as phase‐correlated methods suffer from under‐sampling artifacts. This was found for a representative clinical data set in terms of clinical characteristics and with a large variation between patients (e.g., presence of metallic objects). However, all patients were treated in the United States (US) at one PT institute, and therefore certain characteristics (e.g., weight, size, age) might differ for patients treated in other countries and/or PT institutes.

This improvement in MA‐ROOSTER compared to PC‐FDK was also in concordance with the findings reported in the SPARE challenge and other recent studies that compared an advanced reconstruction method to the conventional PC‐FDK method.^4^, ^13^, ^15^ The necessity of applying an advanced reconstruction method as the MA‐ROOSTER method was further demonstrated through deformable contour warping of the GTVs. GTV centroid motion amplitudes based on MA‐ROOSTER reconstructions showed to be the most realistic.

One limitation is the absence of a ground truth to verify the exact daily motion. This could have been done using advanced techniques such as soft tissue tracking, or to simulate CBCT data including a ground truth motion. In a clinical setting, however, the GTV motion will be relatively compared with the motion found during treatment simulation. The MA‐ROOSTER reconstructed images showed sufficient image quality to be employed for this task, in contrary to the PC‐FDK reconstructions.

It was shown that the ANACONDA algorithm handles CT‐CBCT data well for contour warping, but we can assume that it is not as accurate as CT‐CT DIR.^21^ However, we consistently used this DIR algorithm for both MA‐ROOSTER and PC‐FDK reconstructions on the same patient data set, thus can compare the two methods with the same DIR accuracy.

Despite that the MA‐ROOSTER found motion was the closest to the range of motion found for the 4DCTs, differences remained as the two modalities differ in image quality. Manual checking of contours in 4DCBCTs after deformable warping could improve the motion estimation. This was not performed for the purpose of this study to objectively compare the image quality and measured motion. Other factors like tumor growth, tumor shrinkage, or pleural effusion could also result in differences in measured motion, especially between the simulation 4DCT and daily 4DCBCTs.

Generally, the image quality should always be evaluated in the context of the image purpose. MA‐ROOSTER reconstructed 4DCBCTs were found suitable in our opinion in the context of tumor motion assessment. For more advanced applications, as for example 4DCBCT‐based proton dose calculations, further improvements will be required to warrant sufficient image quality.^17^ The finite range of protons and their sensitivity to changes in water equivalent thickness make it important to monitor variations in motion of the tumor and surrounding tissue. Especially when variations occur in the beam paths, at the distal edge or the lateral penumbra regions. High‐quality 4DCBCT reconstructions will therefore be of interest to monitor the anatomical and motion differences accurately.

The reconstruction workflow used for this research is a semi‐automatic workflow. We found an improved performance when visually checking and manually adjusting the otherwise automatically selected maxima and minima of the extracted respiratory signal during the phase binning process. The improvement was small which might justify skipping this manual intervention in favor of a fully automated workflow. Future work will investigate alternative methods for improved automatic phase binning, for example employ an external breathing signal.

Currently, the MA‐ROOSTER workflow takes up to 40 min, which includes a 15–20 min preparation phase, during which the deformation vector fields between the 4DCT phases are computed as prior motion information. This preparation could also be done offline in advance. However, even the remaining 15–20 min for the 4DCBCT reconstruction itself are currently not suitable for an online employment. That means that 4DCBCT images will at first only be available for offline motion monitoring.

Further steps will be taken to improve the MA‐ROOSTER reconstructions for motion monitoring and our future aim would be to implement MA‐ROOSTER in the clinic.

## 5 CONCLUSION

The MA‐ROOSTER reconstruction method performed well in terms of 4DCBCT image quality and GTV motion assessment. This was shown for a multifaceted, representative (N)SCLC patient data set acquired at a scanned proton gantry. MA‐ROOSTER 4DCBCTs appear to be promising for daily 4DCBCT motion monitoring as part of a future adaptive PT workflow.

## CONFLICT OF INTEREST

All the co‐authors have no conflict of interest to disclose.
